# A Lightweight Channel–Frequency Joint Attention Network for Underwater Ship-Radiated Noise Separation

**DOI:** 10.3390/s26206382

**Published:** 2026-10-09

**Authors:** Junxian Dong, Qiong Yang, Ying Li

**Affiliations:** School of Computer Science, Xi’an Polytechnic University, Xi’an 710600, China

**Keywords:** underwater acoustic signals, end-to-end source separation network, attention mechanism, DPRNN, multi-scale TCN

## Abstract

Underwater acoustic signals (UAS) in marine environments are often contaminated by noise from biological activity, ocean waves, and equipment. Under low signal-to-noise ratio (SNR) conditions, target ship-radiated components and background noise are strongly mixed, making reliable underwater detection, recognition, and communication challenging. To address this problem, this paper proposes Multi-Scale TCN, DPRNN, and Attention TasNet (MSDA-TasNet), a lightweight end-to-end source separation network for underwater ship-radiated noise. The proposed method combines time-domain convolutional encoding, a dual-path recurrent neural network (DPRNN), and a multi-scale temporal convolutional network (multi-scale TCN) to model both short-term details and long-term dependencies of UAS. To further enhance target-aware feature representation, this paper designs a novel channel–frequency attention mechanism that adaptively emphasizes target-related spectral cues. Experiments on two underwater acoustic datasets show that MSDA-TasNet achieves better separation performance than several representative baselines in terms of Signal-to-Distortion Ratio (SDR), Scale-Invariant Signal-to-Distortion Ratio (SI-SDR), SDR improvement (SDRi), SI-SDR improvement (SI-SDRi), and Segmental Signal-to-Distortion Ratio (SegSDR). The results indicate that the proposed network can improve target signal recovery under complex underwater acoustic noise while maintaining a relatively low parameter count and computational cost.

## 1. Introduction

Underwater acoustic signals support a wide range of applications, including marine environmental monitoring, underwater target detection, long-range underwater communication, and intelligent target recognition. At low signal-to-noise ratios (SNRs), strong background interference can mask weak target components and obscure their temporal and spectral characteristics, thereby reducing the reliability of subsequent detection, localization, recognition, and other underwater acoustic applications. In particular, ship-radiated noise contains operating-condition-related structures, such as harmonic components and modulation patterns. Recovering these target-related characteristics while suppressing strong background interference is therefore an important problem in underwater acoustic signal enhancement and source separation.

Traditional underwater acoustic enhancement and separation methods generally take a noisy waveform, a multichannel observation, or an estimate of the noise and reference signal as input, and produce an enhanced waveform, an estimated spectrum, or separated source signals as output. According to the number of sensors, these methods can be broadly divided into single-channel and multi-channel approaches. Single-channel methods include spectral subtraction, Wiener filtering, and adaptive filtering. Spectral subtraction estimates the noise spectrum and attenuates the corresponding components in the observed signal, providing a simple and computationally efficient solution [1,2]. However, inaccurate noise estimation may leave residual noise or introduce artificial spectral artifacts. Wiener filtering estimates the statistical properties of the signal and noise and derives an enhanced signal according to the minimum mean square error criterion [3,4]. It can provide effective noise suppression under relatively stable conditions, but its performance may degrade when the noise statistics vary rapidly. Adaptive filtering updates the filter parameters according to the observed signal and can therefore adapt to time-varying interference [5,6]. Nevertheless, its performance depends on parameter selection, convergence and stability, and the availability of a suitable reference signal. Multi-channel methods exploit spatial information provided by sensor arrays. Subspace-based methods separate signal and noise according to their estimated statistical subspaces, which are usually derived from multichannel covariance information [7,8]. Blind source separation (BSS) methods recover individual sources from their mixtures according to statistical properties, with independent component analysis (ICA) being a representative approach [9]. These methods can exploit spatial or statistical information, but their performance may depend on array configuration, source independence, environmental conditions, and the number of available sensors. Consequently, traditional approaches can be effective when their signal and noise assumptions are appropriate, but their adaptability may be limited in complex and non-stationary underwater acoustic environments.

These limitations have motivated the development of machine learning and deep learning methods for underwater acoustic enhancement and source separation. Traditional methods rely on manually specified signal models, noise statistics, reference signals, or array assumptions, whereas learning-based methods attempt to learn a nonlinear mapping directly from training data [10,11]. Depending on the network architecture and training objective, the input can be a raw time-domain waveform, a time–frequency representation, or another acoustic feature, while the output can be an enhanced waveform, a separation mask, an embedding, or an estimated source signal. Existing studies may use simulated mixtures, controlled recordings, real-world recordings, or combinations of these data sources. In addition, the target and interference definitions, SNR ranges, and evaluation metrics can vary substantially across studies, which means that reported performance cannot always be compared directly. Early convolutional neural network (CNN)-based approaches mainly extracted local structural features from acoustic representations and demonstrated the potential of deep learning for noise suppression and source separation [12]. Discriminative embedding methods such as Deep Clustering further showed that neural networks can learn representations for grouping different signal components in complex acoustic scenes [13]. However, standard CNNs rely primarily on local convolutional operations. Capturing long-range temporal dependencies and global spectral relationships may therefore require deeper networks, larger receptive fields, or additional modeling mechanisms [14,15]. Recurrent neural networks (RNNs) and their variants, including long short-term memory (LSTM) and gated recurrent unit (GRU) networks, were introduced to model temporal dependencies in sequential waveform or feature representations [16,17]. These models can incorporate information from longer temporal contexts, but their sequential computation limits parallelism and may reduce computational efficiency for long sequences. Dual-path recurrent neural networks (DPRNNs) address this limitation by dividing a long sequence into shorter segments and alternately modeling intra-segment and inter-segment dependencies [18]. This design enables the network to capture both local details and long-term temporal relationships. Temporal convolutional networks (TCNs), in contrast, use dilated convolutions to enlarge the receptive field while maintaining parallel computation [19,20]. Multi-scale TCN structures further introduce convolutional operations at different temporal scales, allowing local and relatively long-range temporal variations to be modeled simultaneously [21,22]. These developments represent a gradual transition from local feature extraction toward joint modeling of short- and long-term temporal dependencies. End-to-end time-domain separation networks provide another important direction for acoustic source separation. TasNet and ConvTasNet use learnable encoder–separator–decoder structures to process time-domain waveforms without relying on a fixed short-time Fourier transform (STFT) representation [20]. The encoder converts the waveform into a learned latent representation, the separator estimates source-related features or masks, and the decoder reconstructs the separated waveform. ConvTasNet has demonstrated strong separation capability and relatively low processing latency by learning a task-dependent representation of the input waveform. However, this representation does not explicitly impose a frequency-domain structure. For underwater ship-radiated noise, target-related spectral distributions and modulation characteristics may provide important discriminative information, especially when weak target components are partially masked by strong background interference [23]. A time-domain separation framework that does not explicitly enhance such characteristics may therefore underuse valuable frequency-related information.

Attention mechanisms provide a possible solution by adaptively reweighting informative feature components. Channel attention assigns different weights to feature channels according to their importance [24], while temporal attention emphasizes informative regions in the temporal dimension and can help model long-range dependencies [25]. For underwater acoustic signals, spectral information is also important because ship-radiated noise may contain characteristic energy distributions, harmonic structures, and modulation patterns. Existing studies have therefore explored the combination of attention mechanisms with frequency-related or modulation features to improve underwater acoustic target representation [26]. Decomposition-based approaches have also been investigated for ship-radiated noise denoising, demonstrating the importance of robust noise suppression in complex marine environments [27,28]. In addition, lightweight attention-based networks have been applied to ship-radiated noise classification, indicating that adaptive feature selection can improve representation while controlling model complexity [29]. However, only a limited number of end-to-end separation frameworks jointly model channel-wise feature importance and frequency-related characteristics within the same separation process.

Recent studies have explored lightweight and multi-scale architectures for underwater acoustic and ship-radiated noise recognition [30,31]. Cross-attention and hybrid attention mechanisms have also been introduced to improve underwater acoustic feature representation [32,33]. In addition, auditory scene analysis-guided deep learning has been investigated for ship-radiated noise separation [34], while data augmentation and time–frequency joint attention have been applied to underwater acoustic communication signal classification [35]. Despite these advances, most existing studies focus on recognition, classification, or communication signal processing, and relatively few investigate single-channel end-to-end separation of ship-radiated noise under low-SNR conditions. Moreover, existing methods do not generally combine channel–frequency feature enhancement with multi-scale temporal modeling and long-range dependency modeling within a lightweight waveform-level separation framework.

Taken together, underwater acoustic enhancement and separation methods have progressively evolved from explicit signal models to data-driven feature learning and end-to-end temporal modeling. Traditional methods can provide effective and computationally efficient solutions when the signal and noise characteristics can be reasonably modeled, but their adaptability may be limited in complex and non-stationary underwater environments. Deep learning methods reduce the dependence on manually specified signal models and provide stronger nonlinear representation capability, while their performance remains affected by training data, noise diversity, SNR coverage, and the mismatch between training and testing conditions. Meanwhile, CNNs, RNNs, TCNs, DPRNNs, and end-to-end time-domain separation networks have progressively improved local feature extraction, temporal dependency modeling, and waveform-level separation. Despite these advances, single-channel end-to-end separation of ship-radiated noise under low-SNR conditions remains challenging. First, existing time-domain separation networks do not always explicitly enhance the spectral characteristics of weak target components. Second, channel-wise and frequency-related features are rarely jointly modeled within the separation process. These limitations indicate the need for a lightweight separation framework that can simultaneously enhance target-related features and capture temporal dependencies at different scales. To address these issues, this paper proposes MSDA-TasNet, a lightweight end-to-end source separation network for underwater ship-radiated noise under low-SNR conditions. The proposed framework follows an encoder–separator–decoder architecture and directly processes mixed waveforms. Within the separation module, a channel–frequency attention mechanism is introduced to adaptively reweight feature channels and frequency-related components, thereby emphasizing target-relevant information and suppressing background interference. In addition, a temporal modeling module combining multi-scale TCNs and a DPRNN is constructed to capture temporal features at different scales and model both local and long-term dependencies. By integrating channel–frequency feature enhancement with multi-scale and dual-path temporal modeling, the proposed method aims to improve the recovery of weak ship-radiated noise while maintaining a relatively lightweight model structure. Experiments are conducted on two underwater acoustic datasets under multiple low-SNR conditions, and the separation performance is evaluated using SDR, SI-SDR, SDRi, SI-SDRi, and SegSDR. The experimental results demonstrate the effectiveness of the proposed method under strong background interference.

The main contributions of this paper are as follows:

1. A channel–frequency attention module is proposed to dynamically reweight feature channels and frequency-related components, thereby enhancing target-relevant information and suppressing background interference.
2. A lightweight MSDA-TasNet architecture is developed by integrating the proposed attention module with multi-scale TCNs and a DPRNN within an encoder–separator–decoder framework. This design improves the modeling of both local and long-term temporal features.
3. Experiments are conducted on two different underwater acoustic datasets under multiple low-SNR conditions. The proposed model is compared with representative baseline methods using several separation metrics, and the results demonstrate its effectiveness in recovering weak ship-radiated noise.

The remainder of this paper is organized as follows. Section 2 describes the proposed methodology and experimental protocol, including the datasets, implementation details, and evaluation metrics. Section 3 reports the experimental results, Section 4 discusses the findings and limitations, and Section 5 concludes the paper.

## 2. Materials and Methods

### 2.1. Overall Model Architecture

**Figure 1:**
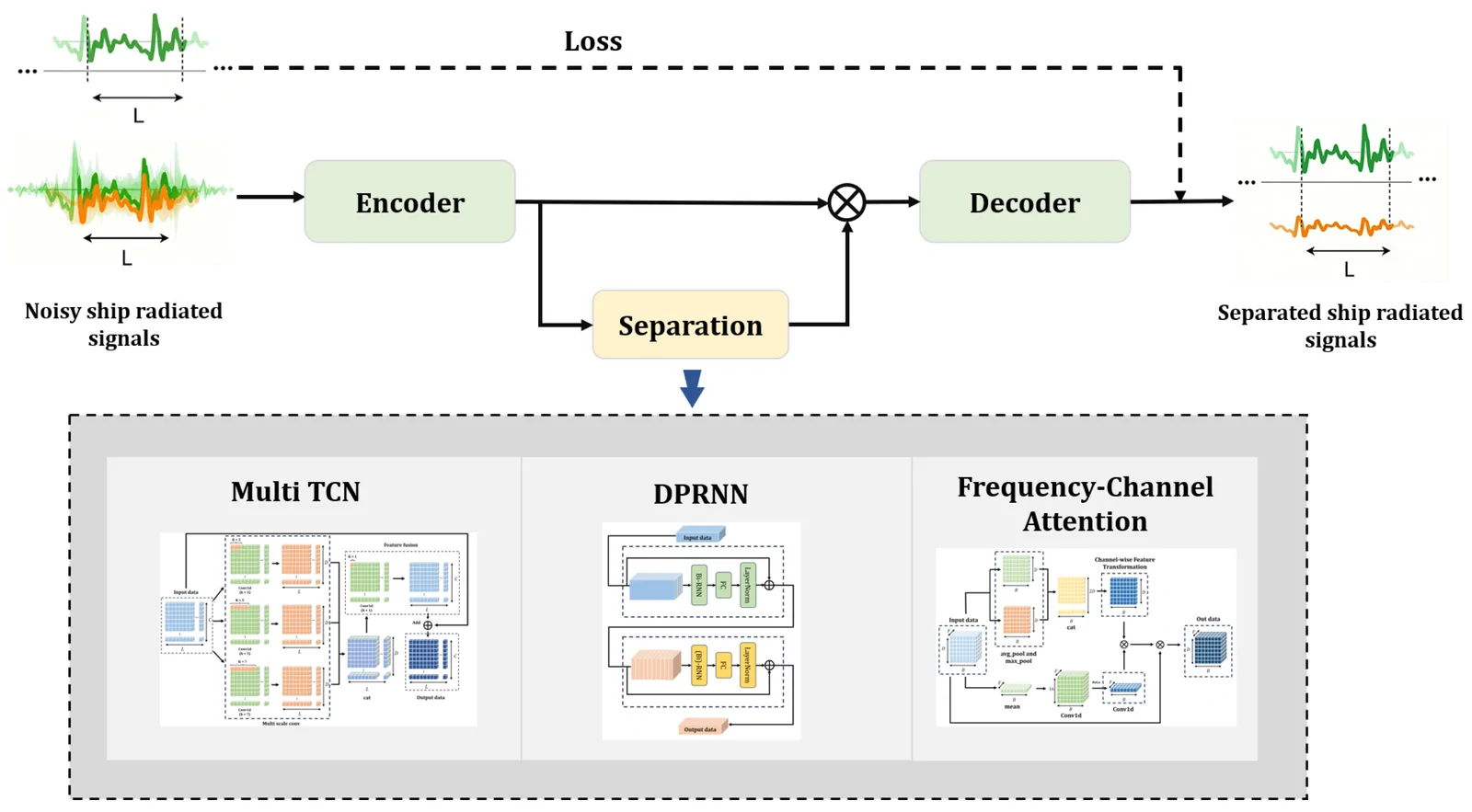
Overall network architecture.

To address the limitations of existing underwater acoustic signal separation methods in modeling temporal dependencies and enhancing target-related frequency features, this paper proposes a lightweight channel–frequency joint attention network, termed MSDA-TasNet, as shown in Figure 1. The model adopts an encoder–separator–decoder architecture and directly processes one-dimensional mixed waveforms, avoiding the information loss and computational cost introduced by explicit time–frequency transformations. In the separation module, a multi-scale TCN captures temporal features at different scales, while DPRNN models long-range dependencies. A channel–frequency attention mechanism is further introduced to suppress noise-related channels and enhance important frequency components of the target signal. Finally, the decoder reconstructs the separated waveform through transposed convolution. The proposed method achieves efficient end-to-end time-domain separation while preserving detailed temporal information under low-SNR conditions.

### 2.2. Encoder–Decoder Architecture

The encoder adopts a one-dimensional convolutional structure to perform time-domain encoding of the input mixed underwater acoustic signal. Let the input mixed signal be *x*(*t*), and the encoding process can be expressed as:

*H* = *Enc*(*x*) = *W_enc_* ∗ *x* (1)

where (*) denotes one-dimensional convolution, *W_enc_* is the learnable filter set, and *H* is the encoded high-dimensional feature representation.

The encoder employs one-dimensional convolution to transform the raw waveform into feature representations while preserving temporal information. These features are then fed into the separation module for source separation. The decoder mirrors the encoder and reconstructs the separated waveform using transposed convolution, which can be expressed as:

(2) $$\hat{x}\left(t\right)=\mathrm{Dec}\left(H\right)$$

where $\hat{x}\left(t\right)$ denotes the reconstructed target underwater acoustic signal, *H* is the latent feature representation from the encoding–separation stage, and *Dec*(∙) represents the decoder mapping implemented by transposed convolution.

**Figure 2:**
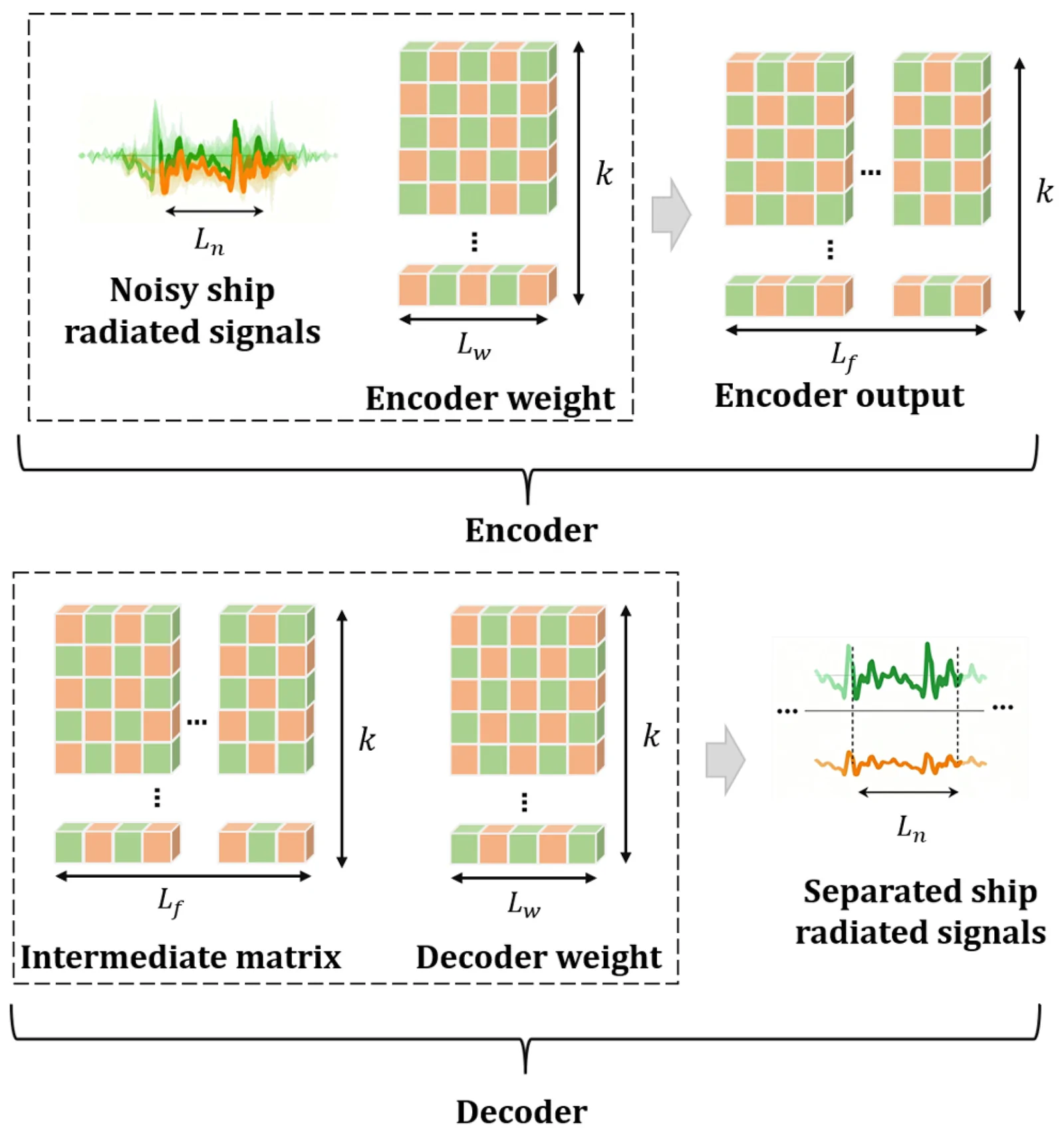
Encoder–decoder architecture. *L_n_* denotes the input/output waveform length, *L_w_* denotes the convolutional filter length, k denotes the number of filters or latent feature channels, and *L_f_* denotes the temporal length of the encoded feature sequence.

Through end-to-end optimization, the encoder and decoder jointly learn representations that are beneficial for reconstructing ship-radiated noise, improving waveform-level accuracy and reconstruction fidelity. Figure 2 shows the encoder–decoder structure in detail.

### 2.3. Separation Module

Underwater acoustic signals contain modulation characteristics at different temporal scales. A temporal convolution with a single receptive field is often insufficient to capture both short-term details and long-term dependencies. To address this problem, a multi-scale temporal convolutional network (multi-scale TCN) is introduced into the separation module. Multiple parallel temporal convolution branches with different kernel sizes are used to extract features at different temporal scales, enabling the network to better model complex underwater acoustic signals. Let the input feature be *X*, and the outputs of different branches are defined as:

*H_k_* = *φ*(*Conv_k_*(*X*))       *k* ∈ {*k*_1_, *k*_2_, ⋯, *k_s_*} (3)

where *Conv_k_*(∙) denotes the one-dimensional convolution with kernel size *k*, and *φ*(∙) represents nonlinear activation and normalization operations.

Features from different scales are fused along the channel dimension, and a residual connection is applied to obtain the final output, which can be expressed as:

*Y* = *Fuse*(*Concat*(*H*_1_, ⋯, *H_s_*)) + *R*(*X*) (4)

where *Concat*(∙) denotes channel-wise concatenation, *Fuse*(∙) represents cross-scale feature fusion implemented by a 1×1 convolution, and *R*(∙) is the residual mapping.

**Figure 3:**
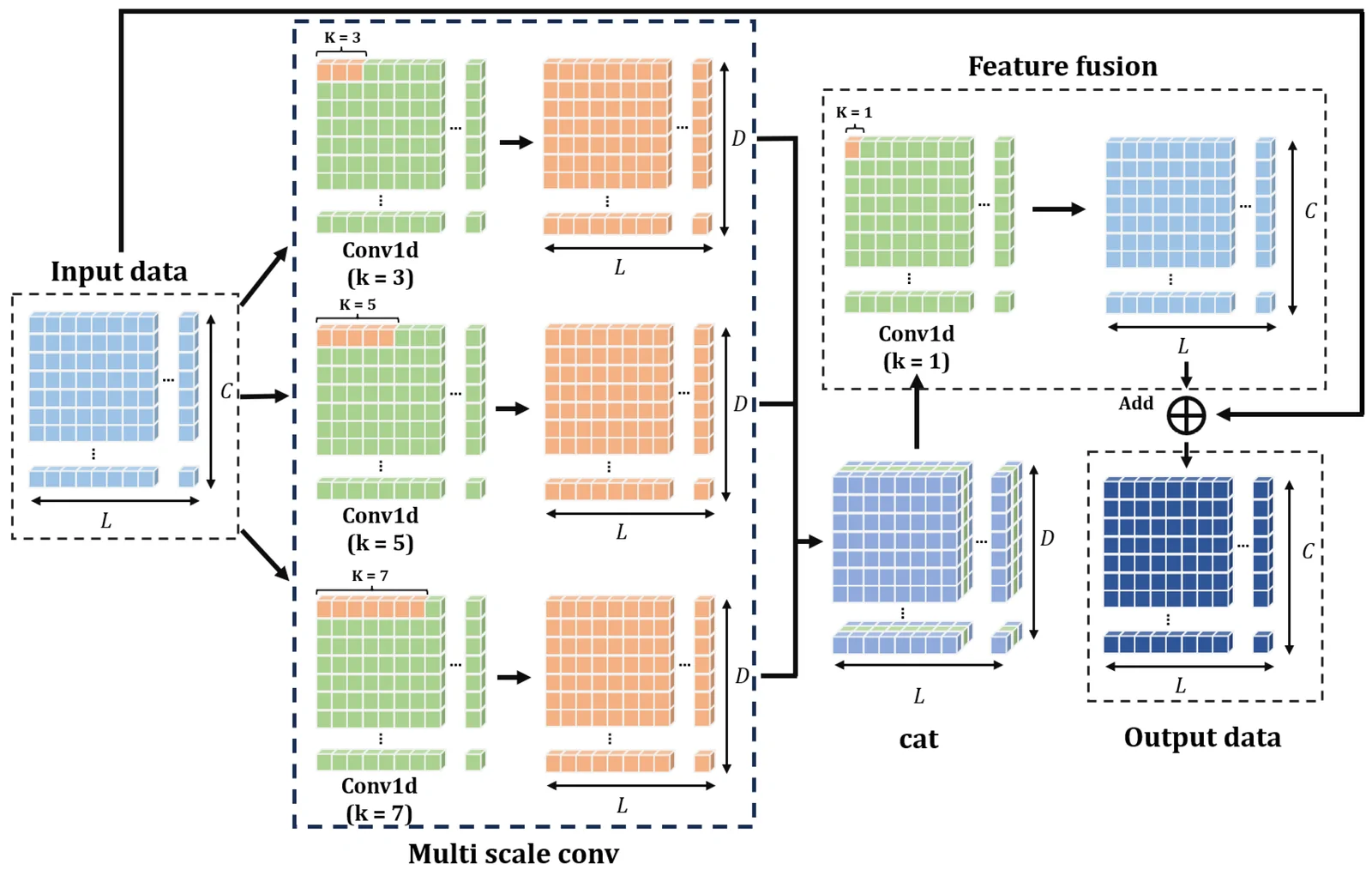
Multi-scale temporal convolutional network. For simplicity, the batch dimension B is omitted from the figure, which illustrates the two-dimensional feature map of a single sample. The input and output feature maps of a single sample both have dimensions *C* × *L*. Here, B, C, D, and L denote the batch size, input and output channel number, channel number of each convolutional branch, and feature sequence length, respectively.

This multi-scale parallel modeling and fusion mechanism can simultaneously capture transient variations and periodic modulation information in ship-radiated noise, thereby enhancing the model’s ability to represent local temporal patterns in non-stationary underwater acoustic signals and providing richer input representations for subsequent temporal modeling. The detailed module structure is shown in Figure 3.

Although the multi-scale TCN can effectively model local temporal features, its receptive field is still limited, making it difficult to capture long-range temporal dependencies in underwater acoustic signals. In complex underwater environments, target signals often exhibit correlations across long time spans that cannot be fully captured by local convolutions alone. To further enhance global temporal modeling, DPRNN is introduced into the separation module. Let the input feature sequence be *X* ∈ ℝ*^B^*^×*T*×*D*^, where B is the batch size, T is the number of temporal steps or segments after reshaping, and D is the feature dimension at each temporal step. DPRNN first models intra-chunk temporal dependencies within local segments using a recurrent network, which can be expressed as:

*H_intra_* = *LN*(*X* + *Proj*(*BiRNN_intra_*(*X*))) (5)

where *BiRNN_intra_*(∙) denotes the intra-chunk bidirectional recurrent neural network (implemented as a bidirectional GRU in this work), *Proj*(∙) represents linear projection with nonlinear transformation, and *LN*(∙) is layer normalization.

On this basis, DPRNN further uses an inter-chunk recurrent network to model global temporal dependencies between different chunks, capturing long-range relationships across time blocks. The process can be expressed as:

*Y* = *LN*(*H_intra_* + *Proj*(*BiRNN_inter_*(*H_intra_*))) (6)

where *BiRNN_inter_*(∙) denotes the inter-chunk bidirectional recurrent network.

**Figure 4:**
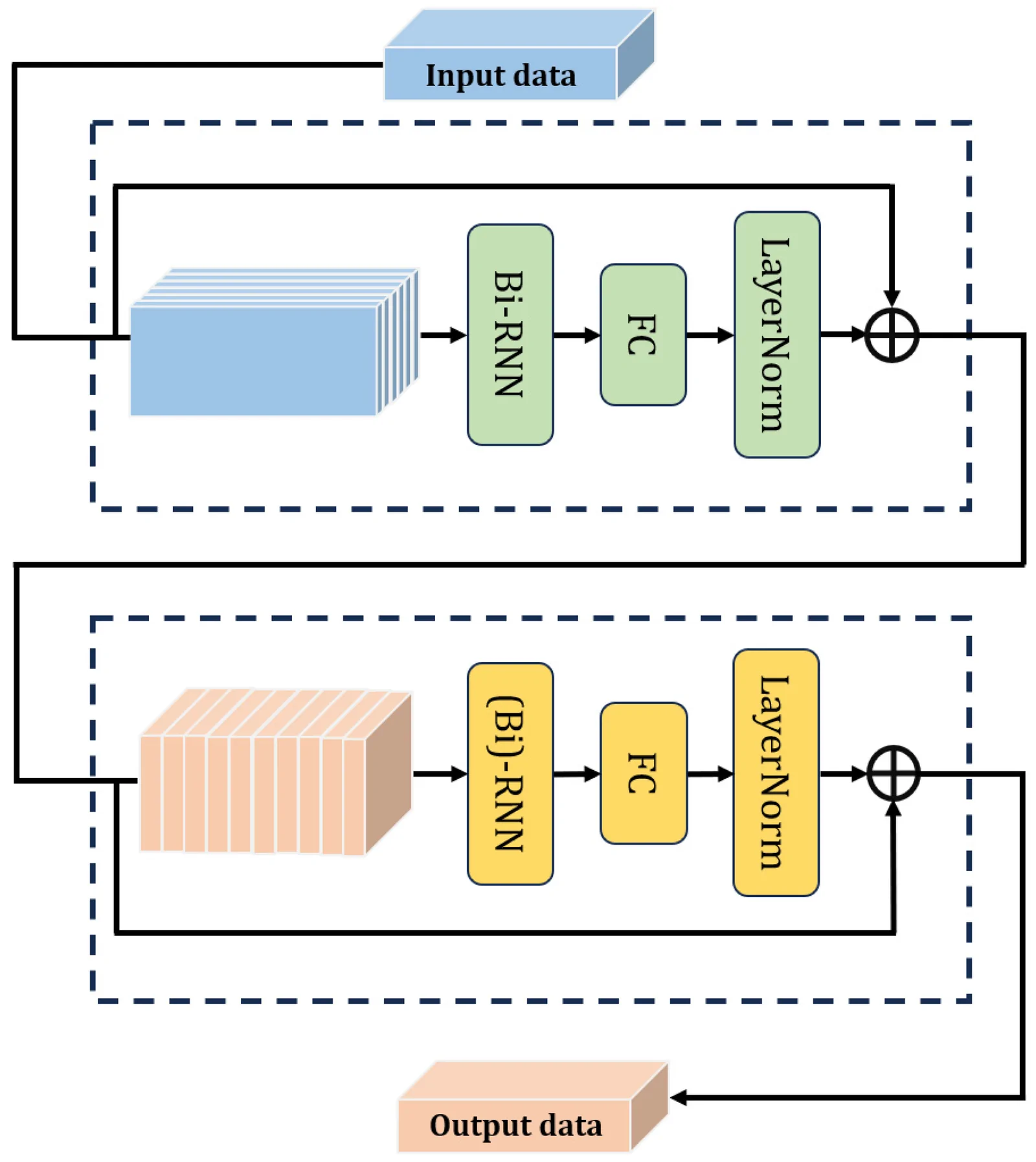
Dual-path recurrent neural network. The input and output feature sequences both have dimensions *B* × *L* × *D*. Here, B, L, and D denote the batch size, encoded feature sequence length, and feature dimension, respectively.

By combining intra-chunk and inter-chunk modeling, DPRNN extends the effective receptive field while maintaining efficiency, enabling it to capture both local details and long-range dependencies in underwater acoustic signals and better represent ship-radiated noise with complex time-varying characteristics. The structure is shown in Figure 4.

In addition, although the proposed framework adopts end-to-end time-domain modeling, underwater acoustic signals still exhibit strong frequency-related characteristics in the feature space. Different ship targets often show distinct energy distributions and dominant frequency structures, which cannot be fully emphasized by time-domain modeling alone. Therefore, a channel–frequency attention mechanism is introduced into the separation module to adaptively recalibrate intermediate features and enhance the representation of target-related frequency bands. Let *X* ∈ ℝ*^B^*^×*D*×*F*^ be the input feature, where B is the batch size, D is the channel dimension, and F is the frequency dimension. Channel attention first uses global average pooling and max pooling to obtain channel statistics and generate channel weights, which can be expressed as:

*A_ch_* = *σ*(*FC*([*AvgPool*(*X*), *MaxPool*(*X*)])) (7)

where *FC*(∙) denotes a nonlinear mapping implemented by fully connected layers, and *σ*(∙) is the sigmoid activation function.

Considering the correspondence between channels and frequency responses in 1D convolution features, a frequency-domain attention mechanism is further introduced. It models the importance distribution along the frequency axis after channel aggregation and is jointly applied with channel attention. The overall attention weighting process can be expressed as:

*Y* = *X* ⊙ (*A_ch_* ∙ *A_freq_*) (8)

where *A_freq_* denotes frequency-domain attention weights generated by a 1D convolution, and ⊙ represents element-wise multiplication.

**Figure 5:**
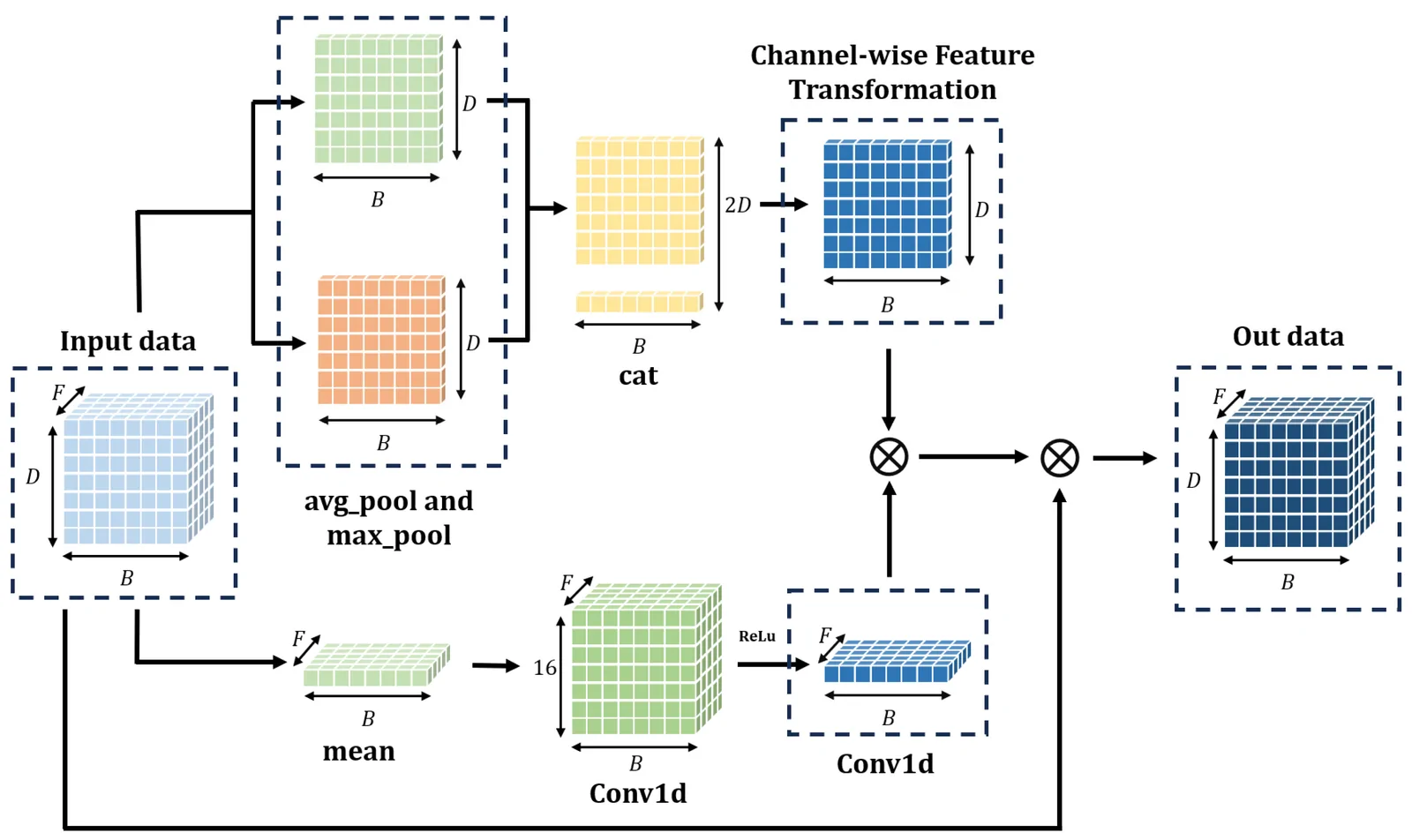
Channel–frequency attention mechanism. The DPRNN output is transposed from *B* × *L* × *D* to *B* × *D* × *F* and used as the input of the attention module. The output also has dimensions *B* × *D* × *F*. Here, B, D, and F denote the batch size, number of feature channels, and frequency dimension, respectively. In this implementation, *F* = *L*.

The structure of the channel–frequency attention module is shown in Figure 5. The module does not use explicit time–frequency transforms, but performs implicit frequency-aware weighting in the time-domain feature space. While keeping the end-to-end framework unchanged, it enhances key components of ship-radiated noise, improving separation performance and robustness in complex noise environments.

In summary, this paper proposes an end-to-end time-domain underwater acoustic source separation method based on an encoder–separator–decoder architecture for direct modeling and reconstruction of mixed signals. In the separation module, multi-scale temporal convolution, dual-path recurrent modeling, and a channel–frequency attention mechanism are integrated to capture local temporal features, long-range dependencies, and frequency-domain discriminative information. Compared to purely time-domain methods, the proposed approach jointly models multi-scale temporal and frequency information, thereby improving feature representation and separation performance in complex underwater acoustic environments.

### 2.4. Loss Function

To jointly constrain the reconstruction quality of the separation results in both time and frequency domains, a composite loss function is adopted, which consists of scale-invariant signal-to-distortion ratio (SI-SDR) loss, multi-scale Short-Time Fourier Transform (STFT) magnitude loss, spectral convergence loss, and waveform-level mean absolute error (MAE) loss. The overall objective is formulated as:

(9) $$\left\{\begin{array}{c} L=\alpha L_{\mathrm{SI}-\mathrm{SDR}}+\beta L_{\mathrm{STFT}}+\gamma L_{\mathrm{SC}}+\delta L_{\mathrm{waveform}}\\ L_{\mathrm{SI}-\mathrm{SDR}}=-10\log_{10}\frac{{\left\|\frac{\langle \hat{x},x\rangle }{{\left\|x\right\|}^{2}}x\right\|}^{2}}{{\left\|\hat{x}-\frac{\langle \hat{x},x\rangle }{{\left\|x\right\|}^{2}}x\right\|}^{2}+\varepsilon }\\ L_{\mathrm{STFT}}=\frac{1}{N}\sum_{i=1}^{N}{\left\|\left|{\mathrm{STFT}}_{i}\left(\hat{x}\right)\right|-\left|{\mathrm{STFT}}_{i}\left(x\right)\right|\right\|}_{1}\\ L_{\mathrm{SC}}=\frac{{\left\|\left|\mathrm{STFT}\left(\hat{x}\right)\right|-\left|\mathrm{STFT}\left(x\right)\right|\right\|}_{F}}{{\left\|\left|\mathrm{STFT}\left(x\right)\right|\right\|}_{F}+\varepsilon }\\ L_{\mathrm{waveform}}=\frac{1}{T}\sum_{t=1}^{T}\left|\hat{x_{t}}-x_{t}\right| \end{array}\right.$$

where *L_SI_*_−*SDR*_, *L_STFT_*, *L_SC_*, and *L_waveform_* denote the SI-SDR loss, multi-scale STFT magnitude loss, spectral convergence loss, and waveform-level mean absolute error loss. The values of α, β, γ, and δ are 1.0, 0.5, 0.3, and 0.2, respectively. $\hat{x}$ and x represent the predicted and target signals. 〈∙,∙〉 denotes the inner product, and ‖∙‖ is the 𝓁_2_ norm. ε is a small constant to avoid division by zero. |*STFT_i_*(∙)| denotes the magnitude spectrum at the i-th scale, where N is the number of scales. ‖∙‖_1_ and ‖∙‖*_F_* denote the L1 norm and Frobenius norm, respectively, and T is the number of time-domain samples.

In this composite loss, the SI-SDR loss constrains overall time-domain consistency, ensuring global structural alignment between the predicted and target signals. The multi-scale STFT magnitude loss enforces constraints at different time–frequency resolutions, improving the modeling of multi-scale spectral structures. The spectral convergence loss reduces the overall difference between predicted and true spectra, preserving spectral shape consistency. The waveform-level mean absolute error loss directly constrains sample-level reconstruction accuracy, improving local detail recovery. The joint optimization of these losses enables the model to maintain both time-domain reconstruction quality and frequency-domain consistency, thereby improving overall ship-radiated noise separation performance.

To evaluate the effectiveness and robustness of the proposed method for ship-radiated noise separation, experiments are designed from three aspects: dataset construction, implementation details, and evaluation metrics. A unified data splitting strategy and consistent settings are used to ensure fair comparisons. Both time-domain and frequency-domain metrics are adopted to comprehensively evaluate the separation performance under complex underwater acoustic conditions.

### 2.5. Dataset Design

Dataset A: The experimental data are constructed from the DeepShip underwater acoustic dataset [36], which contains 613 recordings with a total duration of approximately 47.07 h, covering four vessel types: cargo ships, passenger ships, tankers, and tugs. The original audio is sampled at 32 kHz and segmented into 15 s clips. Ship-radiated noise is used as the target signal, and Gaussian white noise with an SNR from −5 to 5 dB is added to generate noisy signals.

Dataset B: The experimental data are constructed from the QiandaoEar22 dataset [37], which contains 10,611 target recordings (approximately 9 h 28 min) and 25,900 background-noise recordings (approximately 21 h 58 min). The dataset was collected in Qiandao Lake and contains multiple underwater targets and complex background noise with strong non-stationarity and wideband characteristics. The UUA subset is used in this study, and noisy signals are generated by mixing target signals and background noise at random SNRs between −5 and 5 dB.

### 2.6. Experimental Configuration

The experiments were conducted using the PyTorch 2.8.0 framework on a single NVIDIA RTX 5090 GPU. All audio data were first resampled to a unified sampling rate of 32 kHz. During training, fixed-length 2 s audio segments were randomly cropped from the original recordings, corresponding to 64,000 sampling points. For audio signals shorter than 2 s, waveform repetition was employed for padding to ensure a consistent input length, followed by peak normalization. The dataset was randomly divided into training and validation sets at a ratio of 8:2. The batch size was set to 2, and the samples were randomly shuffled during training. To reduce additional memory overhead during small-batch training and improve training stability, data loading was performed using a single process (num_workers = 0), while pinned memory was disabled (pin_memory = False). The random seed was fixed at 42. The model adopts a lightweight encoder–separator–decoder architecture. The encoder uses one-dimensional convolution for feature encoding, with a kernel size of 32, a stride of 16, and an encoded feature dimension of 192. The separation layer consists of three multi-scale TCN layers and three DPRNN modules. The multi-scale TCN employs convolutional kernels with sizes of 3, 5, and 7 to extract local features at different temporal scales. The DPRNN is further used to model long-range dependencies in the sequence. Meanwhile, a channel–frequency joint attention mechanism is introduced to adaptively recalibrate the extracted features, thereby enhancing target-related features and suppressing interference information. The entire network contains approximately 3.4M trainable parameters, reducing model complexity while maintaining sufficient feature modeling capability. The model was trained using the AdamW optimizer with an initial learning rate of 2 × 10^−4^ and a weight decay coefficient of 1 × 10^−5^. A StepLR learning rate decay strategy was adopted, where the learning rate was multiplied by 0.9 every 10 epochs. Training was performed for a maximum of 100 epochs, with gradient clipping using a maximum gradient norm of 0.5 and an early stopping strategy with patience set to 10 epochs to improve training stability and prevent overtraining. At the end of each epoch, the model was evaluated on the validation set, and the model achieving the best validation SDR was saved as the final model. The average wall-clock training time was approximately 6 min per epoch on the NVIDIA RTX 5090 GPU.

The above experimental settings were kept consistent across all experiments. To comprehensively evaluate the underwater acoustic signal separation performance of the proposed MSDA-TasNet, experiments were conducted on two underwater acoustic datasets and compared with representative baseline models. In addition, ablation experiments were performed to further analyze the contributions of the key modules. All experiments used the same experimental configurations and evaluation metrics to minimize the influence of experimental variations and ensure fair, comparable, and reproducible results.

### 2.7. Performance Evaluation Metrics

To comprehensively evaluate the proposed method for ship-radiated noise separation, five complementary metrics are adopted: Signal-to-Distortion Ratio (SDR), Scale-Invariant Signal-to-Distortion Ratio (SI-SDR), SDR improvement (SDRi), SI-SDR improvement (SI-SDRi), and Segmental Signal-to-Distortion Ratio (SegSDR). These metrics are selected to assess different aspects of separation performance, including global reconstruction quality, scale-invariant separation quality, improvement over the noisy mixture, and segment-level temporal stability. Let *x* denote the target signal and $\hat{x}$ denote the separated signal. The SDR is defined as:

(10) $$\mathrm{SDR}=10\log_{10}\frac{{\left\|x\right\|}^{2}}{{\left\|\hat{x}-x\right\|}^{2}}$$

Based on this, SDRi is defined as the difference in SDR between the separated signal and the original noisy mixture, i.e.:

*SDRi* = *SDR_out_* − *SDR_in_* (11)

This metric directly reflects the model’s improvement in suppressing noise and interference. To address the sensitivity of SDR to signal scaling, the scale-invariant SDR (SI-SDR) is introduced, defined as:

(12) SI-SDR=10 log10αx2x^− αx2 , α=x^Txx2

where α is the optimal scaling factor used to linearly scale the target signal *x* to best match the estimated signal $\hat{x}$. Accordingly, SI-SDRi measures the performance improvement in the model in a scale-invariant manner:

SI-SDRI = SISDRI*_out_* − SISDRI*_in_* (13)

To further evaluate the model’s local temporal stability, SegSDR is introduced. It divides the signal into K short segments, computes SDR for each segment, and then averages the results, defined as:

(14) $$\mathrm{SegSDR}=\;\frac{1}{K}\sum_{k=1}^{K}10\log_{10}\frac{{\left\|x_{k}\right\|}^{2}}{{\left\|{\hat{x}}_{k}-\;x_{k}\right\|}^{2}}$$

where *x* and $\hat{x}$ denote the target signal and the estimated signal, respectively. *SDR_in_* and *SDR_out_* represent the SDR values of the mixed signal and the separated signal. *x_k_* and ${\hat{x}}_{k}$ denote the signals in the k-th segment, where *k* is the number of segments, and ‖∙‖ represents the *l*_2_ norm. By jointly analyzing these metrics, the model’s separation performance and robustness in complex underwater acoustic environments can be comprehensively evaluated from overall reconstruction quality, relative improvement, and temporal consistency.

## 3. Results

This section reports the quantitative comparison, ablation, visualization, and computational results for the proposed method. 

### 3.1. Comparative Experiments

**Figure 6:**
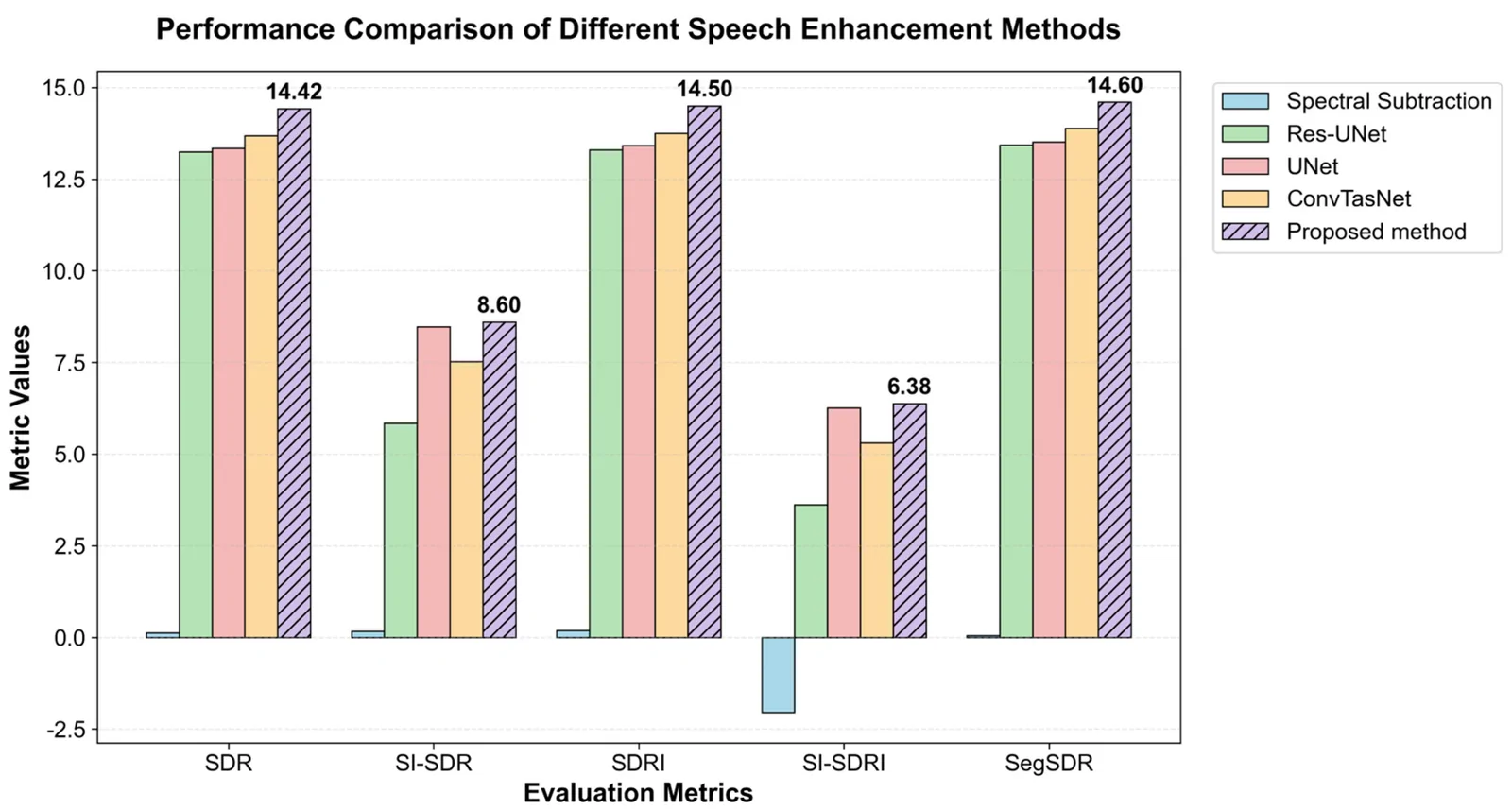
Comparison results of different methods on Dataset A.

To verify the effectiveness of the proposed method, spectral subtraction, UNet [38], Res-UNet [39], and ConvTasNet [20] were selected for comparison. Five metrics, including SDR, SI-SDR, SDRi, SI-SDRi, and SegSDR, were evaluated on two underwater acoustic datasets. The results on Dataset A are shown in Figure 6.

Spectral subtraction performed the worst, with SDR and SI-SDR values of only 0.12 dB and 0.17 dB, respectively, while its SI-SDRi was −2.05 dB, indicating limited ability to suppress complex underwater noise and recover the target signal. Res-UNet, UNet, and ConvTasNet achieved much better performance. ConvTasNet obtained an SDR of 13.68 dB and a SegSDR of 13.89 dB, outperforming Res-UNet overall, although its SI-SDR and SI-SDRi were lower than those of UNet. The proposed method achieved the best results on all five metrics, with SDR, SI-SDR, SDRi, SI-SDRi, and SegSDR values of 14.42 dB, 8.60 dB, 14.50 dB, 6.38 dB, and 14.60 dB, respectively. Compared to the second-best method, SDR and SegSDR were improved by 0.74 dB and 0.71 dB, demonstrating better underwater noise suppression and target signal preservation.

Compared to Dataset A, Dataset B contains more complex and non-stationary background noise, leading to performance degradation across all models. The results are shown in Table 1.

### 3.2. Ablation Study

To evaluate the contribution of each module to underwater acoustic signal separation performance, ablation experiments are conducted on Dataset A. The baseline model is compared to three variants obtained by progressively incorporating the multi-scale TCN, DPRNN, and channel–frequency attention module. All models are evaluated under the same experimental settings using SDR, SI-SDR, SDRi, SI-SDRi, and SegSDR, allowing the contribution of each module to be assessed quantitatively.

**Figure 7:**
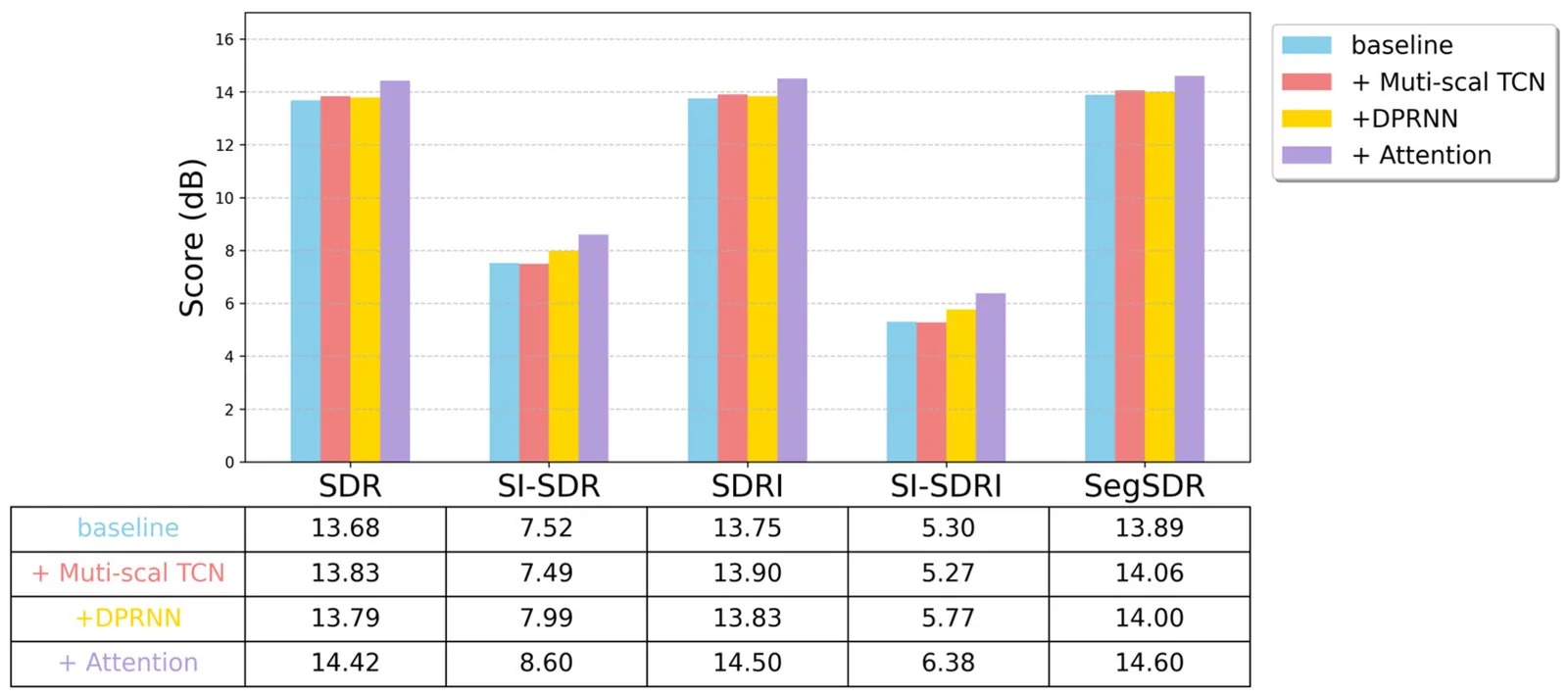
Ablation study results.

As shown in Figure 7, introducing the multi-scale TCN improves local feature extraction, while DPRNN is effective in modeling long-term temporal dependencies. The channel–frequency attention module significantly enhances target frequency bands and suppresses noise components. The full model, combining all modules, achieves the best performance across SDR, SI-SDR, SDRi, SI-SDRi, and SegSDR, fully demonstrating the complementarity of the proposed components and the effectiveness of the overall design.

### 3.3. Visualization

To visually compare underwater acoustic separation and noise suppression performance, spectrograms of the noisy mixture, clean target signal, and enhanced results from different models are analyzed on Dataset A.

**Figure 8:**
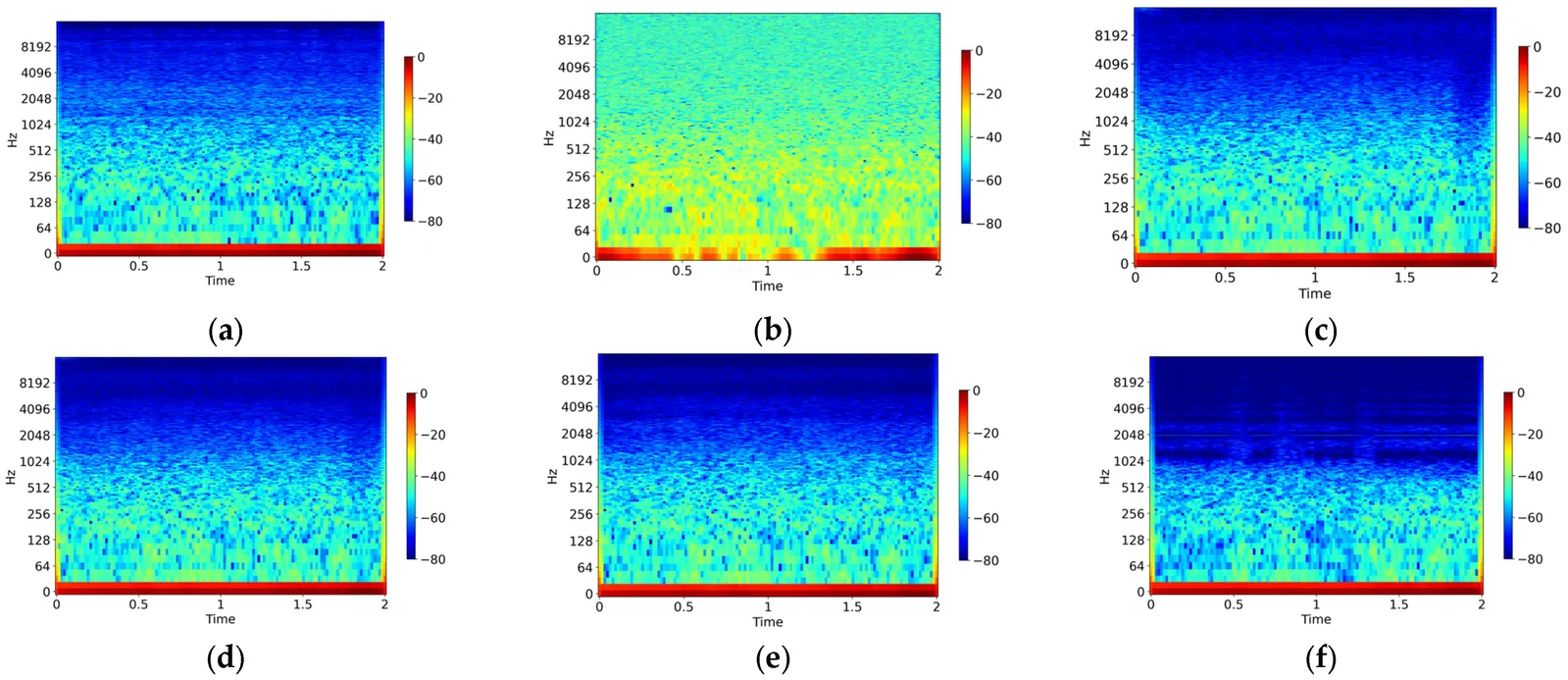
Time–frequency spectrograms of the clean target signal, the noisy underwater acoustic mixture, and the separated results: (**a**) original clean underwater acoustic signal; (**b**) noisy mixed underwater acoustic signal; (**c**) signal separated by the proposed method; (**d**) signal separated by ConvTasNet; (**e**) signal separated by Res-UNet and (**f**) signal separated by UNet.

As shown in Figure 8, the noisy mixture (Figure 8b) contains strong broadband interference, particularly in the high-frequency region, which obscures the harmonic structure of the clean target signal (Figure 8a). All four separation methods reduce a substantial portion of the background interference and recover the main low- and mid-frequency components. The proposed method (Figure 8c) produces the clearest and most continuous harmonic structures, with relatively little residual high-frequency noise and better preservation of target-related spectral details. ConvTasNet (Figure 8d) also achieves effective noise suppression and preserves the main spectral components, but its recovered harmonic structures are less distinct than those of the proposed method. Res-UNet (Figure 8e) and UNet (Figure 8f) remove part of the interference but retain more residual noise and exhibit weaker or less continuous harmonic patterns. Overall, the proposed method provides the best balance between broadband noise suppression and preservation of the target signal’s spectral structure.

To characterize performance distribution differences among methods, we conduct separation experiments on selected validation samples from Dataset A and evaluate them using SDR, SI-SDR, SDRi, and SI-SDRi.

**Figure 9:**
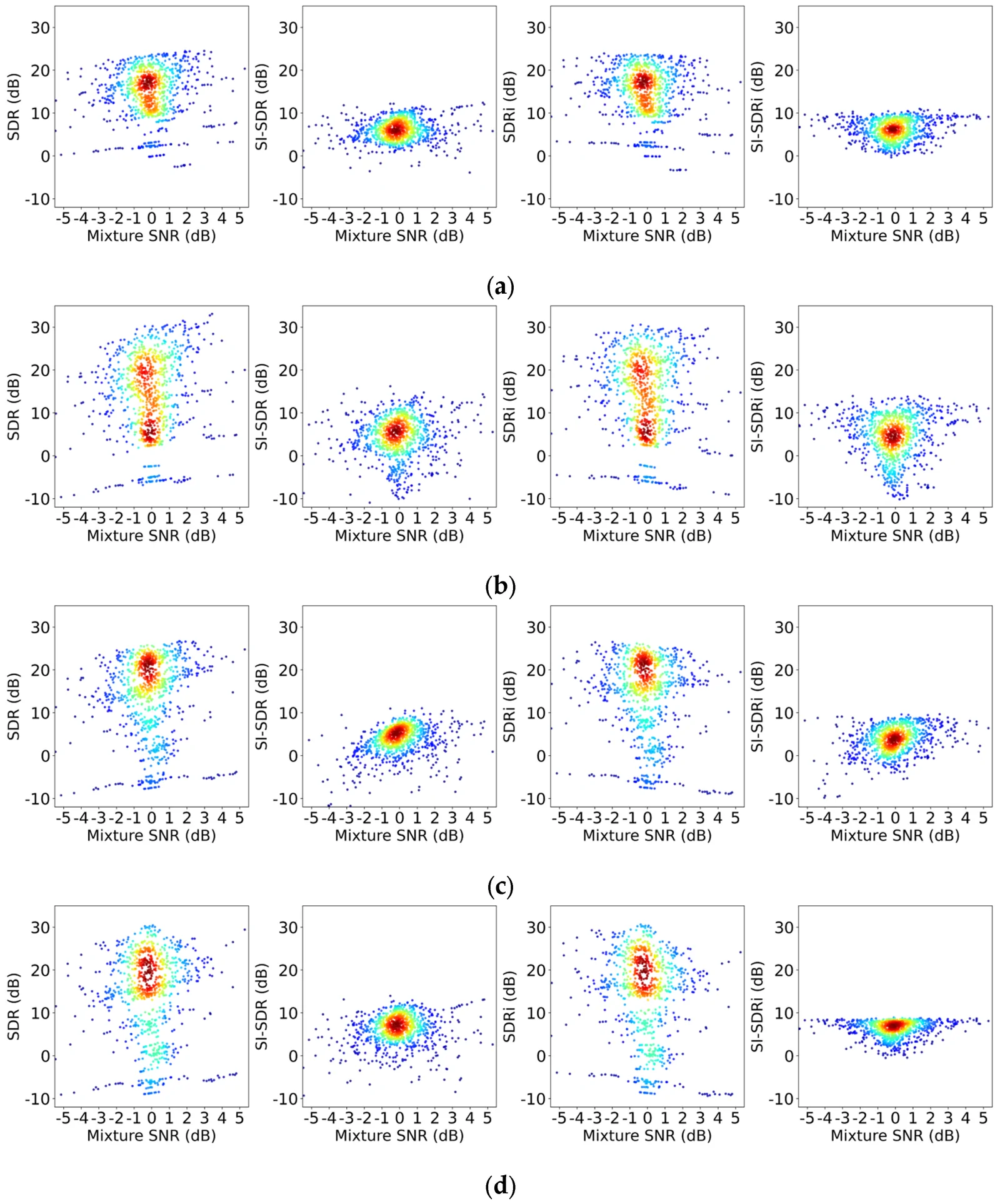
Scatter distributions of different methods on Dataset A in terms of SDR, SI-SDR, SDRi, and SI-SDRi: (**a**) proposed method; (**b**) ConvTasNet; (**c**) Res-UNet; (**d**) UNet.

Figure 9 presents the sample density distributions of SDR, SI-SDR, SDRi, and SI-SDRi for the proposed method, ConvTasNet, Res-UNet, and UNet under different mixture SNR conditions. UNet and ConvTasNet exhibit relatively dispersed metric distributions under low-SNR and strong-interference conditions, with a considerable number of negative-gain samples, indicating less stable separation performance. Res-UNet shows improved overall performance compared with the two models, but its separation gains still exhibit noticeable degradation under strong interference. In contrast, the proposed method produces more concentrated distributions across all metrics, with substantially fewer abnormal negative-gain samples, and maintains relatively stable positive separation gains over the mixture SNR range of −5 to 5 dB. These results indicate that the proposed method provides better separation stability and robustness under low-SNR conditions with strong non-stationary interference.

### 3.4. Computational Efficiency

To verify the advantages of the proposed method in model complexity and computational efficiency, we compare it in terms of parameter size and computational cost.

As shown in Table 2, the proposed method has a model size of 3.4 M parameters, which is substantially smaller than Res-UNet (12.2 M) and UNet (10.8 M), while being comparable to the ConvTasNet baseline (3.45 M). In terms of computational cost, the proposed method requires only 11.38 GFLOPs, which is lower than the ConvTasNet (29.57 GFLOPs), Res-UNet (30.68 GFLOPs), and UNet (45.84 GFLOPs). Compared to the simplified ConvTasNet, Res-UNet, and UNet, the computational cost is reduced by approximately 62%, 63%, and 75%, respectively. These results demonstrate that the proposed method achieves a lower computational cost while maintaining a compact model size, indicating its potential for resource-constrained underwater acoustic applications.

## 4. Discussion

The results show that MSDA-TasNet provides a consistent balance between separation quality and computational efficiency. It achieves the strongest scores on both datasets, with a larger relative advantage on Dataset B, where the background noise is more non-stationary. This suggests that the combination of multi-scale temporal modeling, long-range dependency modeling, and channel–frequency reweighting improves robustness to complex underwater interference.

The ablation and visualization results provide complementary evidence for the contribution of the proposed components: multi-scale TCN strengthens local feature extraction, DPRNN captures long-term dependencies, and the channel–frequency attention module preserves target-related spectral structures. Together with the lower parameter count and GFLOPs, these results support the practical use of the model in resource-constrained underwater acoustic applications. Nevertheless, the current evaluation is limited to two datasets and single-target mixtures, which motivates future validation under broader sea states, stronger interference, and multi-target conditions.

## 5. Conclusions

This study proposed MSDA-TasNet, an end-to-end time-domain network that combines multi-scale TCN, DPRNN, and channel–frequency attention for underwater ship-radiated noise separation. Experiments on the DeepShip and QiandaoEar22 datasets showed that it consistently outperformed the comparison methods on the main separation metrics while requiring fewer parameters and lower computational cost. These results support its effectiveness for weak-target recovery under low-SNR and non-stationary noise. Future work will evaluate broader sea states, stronger interference, and multi-target mixtures.

## Figures and Tables

**Table 1:** Comparison results of different methods on Dataset B.

Model	SDR	SI-SDR	SDRi	SI-SDRi	SegSDR
Res-UNet	5.95 dB	4.64 dB	2.77 dB	2.27 dB	5.04 dB
UNet	5.77 dB	4.37 dB	2.60 dB	2.00 dB	4.80 dB
ConvTasNet	6.62 dB	5.62 dB	3.44 dB	3.25 dB	5.95 dB
**Proposed method**	**7.96 dB**	**6.98 dB**	**4.78 dB**	**4.61 dB**	**7.00 dB**

ConvTasNet outperformed Res-UNet and UNet overall, achieving SDR, SI-SDR, and SegSDR values of 6.62 dB, 5.62 dB, and 5.95 dB, respectively. The proposed method still achieved the best results on all five metrics, with SDR and SI-SDR values of 7.96 dB and 6.98 dB, improving upon ConvTasNet by 1.34 dB and 1.36 dB, respectively. These results demonstrate the stronger robustness and generalization ability of the proposed method under complex noise conditions.

**Table 2:** Model size and GFLOPs for different models.

Model	Model Size (M)	GFLOPs
Res-UNet	12.2	30.68
UNet	10.8	45.84
ConvTasNet	3.45	29.57
**Proposed method**	**3.4**	**11.38**

## Data Availability

The datasets used and/or analyzed during the current study are available from the corresponding author on reasonable request.
